# Comparing structural fingerprints using a literature-based similarity benchmark

**DOI:** 10.1186/s13321-016-0148-0

**Published:** 2016-07-05

**Authors:** Noel M. O’Boyle, Roger A. Sayle

**Affiliations:** NextMove Software, Innovation Centre, Cambridge Science Park, Milton Road, Cambridge, CB4 0EY UK

**Keywords:** Similarity searching, Molecular fingerprints, Structural similarity, Similarity benchmark

## Abstract

**Background:**

The concept of molecular similarity is one of the central ideas in cheminformatics, despite the fact that it is ill-defined and rather difficult to assess objectively. Here we propose a practical definition of molecular similarity in the context of drug discovery: molecules A and B are similar if a medicinal chemist would be likely to synthesise and test them around the same time as part of the same medicinal chemistry program. The attraction of such a definition is that it matches one of the key uses of similarity measures in early-stage drug discovery. If we make the assumption that molecules in the same compound activity table in a medicinal chemistry paper were considered similar by the authors of the paper, we can create a dataset of similar molecules from the medicinal chemistry literature. Furthermore, molecules with decreasing levels of similarity to a reference can be found by either ordering molecules in an activity table by their activity, or by considering activity tables in different papers which have at least one molecule in common.

**Results:**

Using this procedure with activity data from ChEMBL, we have created two benchmark datasets for structural similarity that can be used to guide the development of improved measures. Compared to similar results from a virtual screen, these benchmarks are an order of magnitude more sensitive to differences between fingerprints both because of their size and because they avoid loss of statistical power due to the use of mean scores or ranks. We measure the performance of 28 different fingerprints on the benchmark sets and compare the results to those from the Riniker and Landrum (J Cheminf 5:26, 2013. doi:10.1186/1758-2946-5-26) ligand-based virtual screening benchmark.

**Conclusions:**

Extended-connectivity fingerprints of diameter 4 and 6 are among the best performing fingerprints when ranking diverse structures by similarity, as is the topological torsion fingerprint. However, when ranking very close analogues, the atom pair fingerprint outperforms the others tested. When ranking diverse structures or carrying out a virtual screen, we find that the performance of the ECFP fingerprints significantly improves if the bit-vector length is increased from 1024 to 16,384.

**Graphical abstract:**
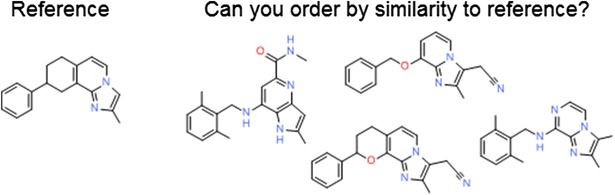
An example series from one of the benchmark datasets. Each fingerprint is assessed on its ability to reproduce a specific series order.

**Electronic supplementary material:**

The online version of this article (doi:10.1186/s13321-016-0148-0) contains supplementary material, which is available to authorized users.

## Background

The Similar Property Principle (SPP) is the observation that structurally similar molecules tend to have similar properties [1]. This is a cornerstone of drug discovery, as it means that successive small changes to the structure of an active should retain biological activity against a target. In this case the SPP is really an expression of the nature of protein–ligand binding. As with any guiding principle, there are exceptions; in drug discovery these are referred to as activity cliffs [2] where small changes in the structure cause large changes in biological activity. Unfortunately the SPP does not provide any guidance on how to identify or measure whether two molecules are structurally similar. Computationally, the most common way to measure this is to compare molecular fingerprints, binary or count vectors that encode features of molecules. This numerical measure of similarity may then be used for similarity searching, ligand-based virtual screens, clustering and diversity analysis [3–5].

As many molecular fingerprints are in widespread use [6], an important question to ask is which are better at measuring structural similarity. Benchmarks for ligand-based virtual screening and investigations of neighbourhood behaviour could be considered as providing an answer to this question. A ligand-based virtual screen [7–11] tests the ability of a similarity measure to identify actives from within a set of decoys given a single active (or a small number of actives) as a query, with the underlying assumption that the actives are more structurally similar to each other than they are to the decoys. In other words, good performance in such screens relies on the reverse of the SPP, that molecules with similar properties are structurally similar. Note that such screens do not consider the degree of similarity beyond the goal of ranking actives higher than decoys. Neighbourhood behaviour studies [12–15] investigate the correspondence between structural similarity measures and similarity in biological space (often a combined measure across several targets). A typical study tries to identify the optimal structural similarity cutoff to yield the best balance of precision versus recall. Note that in this context, a “false similar” pair of molecules is structurally similar but not similar in terms of biological activity.

Several ligand-based virtual screening studies have found that the performance of the ECFP4 fingerprint is either the best or among the best, although one should note that the majority of these studies used a small number of targets (11 or fewer) and lacked an analysis of statistical significance. Hert et al. [16] found that ECFP4 fingerprints performed best on average for 11 targets from the MDDR (a viewpoint supported by a re-analysis of the same dataset by Bender et al. [17]). Sastry et al. [18] also looked at 11 targets from the MDDR and found that a radial fingerprint (which they identify as synonymous with ECFP, although different atom types may be used) was among the top three (Molprint2D and a dendritic fingerprint were better). However, on the Briem and Lessel benchmark dataset [19] (5 targets from the MDDR), Duan et al. [20] found that the same radial fingerprint (that is, from Sastry et al.) was best although Molprint2D was close in performance. In contrast to the previous studies, the 2013 study by Riniker and Landrum included 88 targets, almost an order of magnitude more than previous ligand-based virtual screening studies, and did include a treatment of statistical significance. While the ECFP4 fingerprint had the best mean rank, the analysis was not able to show its mean rank to be significantly better than that of topological torsions, ECFP6 or ECFC4. In the field of neighbourhood behaviour, Papadatos et al. [14] found that ECFP6, SEFP4 and SEFP6 performed consistently well across 27 chemotype-based datasets covering 9 targets.

Here we describe a new type of benchmark for structural similarity that takes into account the fact that structural similarity is a continuous scale rather than a binary property. This is not a virtual screen but instead consists of series of molecules arranged by structural similarity with respect to a reference.

While the SPP does not provide any guidance on how to rank molecules by structural similarity, as a starting point we consider molecule A and B to be similar if it is reasonable that a medicinal chemist would synthesise and test A and B around the same time as part of the same medicinal chemistry program. This definition has a number of attractive features. First of all, it is a measure of similarity directly linked to the task in which we are interested. It is also widely used in practice; typically a medicinal chemist has the final say on which molecules are selected for synthesis/testing from a set of hits in a virtual screen.

While there has been some recent work on comparing human and computational measures of structural similarity [21], it is still the case that the “cognitive algorithms by which medicinal chemists perceive similarity are largely unknown” (to quote Maggiora et al. [22]). Our approach is to use the co-occurrence of A and B in an activity table in the published medicinal chemistry literature as an indication that two molecules are similar according to this definition, information which is available from the ChEMBL database [23]. In this way we extract pairs of molecules which were considered similar enough to be part of the same medicinal chemistry project, and on which a medicinal chemist was willing to bet their time that both would be active against the same target.

It may be that the answer to the question posed in the title depends on the degree of similarity. That is, which fingerprint is best may be different when searching for close analogues versus searching for more distant analogues, versus separating actives and decoys in a virtual screen of compounds available for purchase. To consider this, we have developed two distinct benchmarks, which test the ability to distinguish similarity within different ranges.

**Fig. 1:**
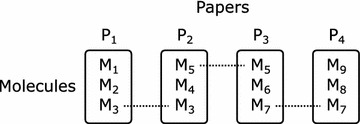
Composition of a series in the multi-assay benchmark. The *diagram* shows a series consisting of five molecules M1, M3, M5, M7 and M9 (in that order) taken from four assays in four different papers, where each assay has a compound in common

1. The single-assay benchmark tests the ability to rank very similar structures relative to a reference. Five molecules differing by about 0.4 log units from each other were selected from the same ChEMBL assay. These are structurally similar according to our definition. Given the most active as the reference, the others were ordered in decreasing order of activity. Our assumption is that the more similar the activity is to the reference, the more similar the structure will be.
2. The multi-assay benchmark tests the ability to rank more diverse structures relative to a reference. Given a reference molecule, a series of four molecules with decreasing similarity (that is, increasing distance) to the reference was generated by linking from one paper with activity data to another through molecules in common between both. Figure 1 illustrates the concept: M1 and M3 are similar according to our definition, as are the pairs M3 and M5, M5 and M7, M7 and M9. We assume that relative to M1, structural similarity will decrease as one moves through the series M3, M5, M7, and M9 due to the size of chemical space (even in the vicinity of a particular target) and the nature of a random walk.

While no one similarity measure will be the best in every instance, the main goal of the current study is to determine which similarity measures in general correspond best to a medicinal chemist’s notion of similarity, and which should be avoided. Furthermore, we wish to provide benchmarks that will aid the development of improved similarity measures as they can distinguish between even small differences in performance. As improvements typically stem from incremental changes and parameter testing, this sensitivity will help guide these efforts. Finally, by comparison with the corresponding results from a re-analysis of the virtual screening study of Riniker and Landrum, we can investigate the extent to which structural similarity is the same at different ranges of similarity, and determine whether the described benchmarks be useful in developing fingerprints with improved performance in a virtual screen.

## Methods

### Structural fingerprints tested

The molecular fingerprints used were taken from the benchmarking platform described by Riniker and Landrum [9] and are listed in Table 1. Although their study focused on results for 14 fingerprints, the associated code [24] includes a further 14, mainly additional variants of circular fingerprints but also hashed forms of atom pairs (HashAP) and topological torsions (HashTT). In this study we have used the full set of 28 fingerprints as implemented in the RDKit version 2015.09.2 [25].

The fingerprints may be classified as follows. Additional details are in the publication by Riniker and Landrum:

1. *Path*-*based fingerprints* RDKx where x is 5, 6, 7 (hashed branched and linear subgraphs up to size x), TT (topological torsion [26], a count vector) and a binary vector form HashTT, AP [27] (atom pair, a count vector) and a binary vector form HashAP.
2. *Substructure keys* Avalon [28], MACCS.
3. *Circular fingerprints* The extended-connectivity fingerprints [29] ECFPx where x is 0, 2, 4, 6, and the corresponding count vectors denoted as ECFCx. Also the feature-class fingerprints FCFPx and corresponding count vectors FCFCx where x is 2, 4, 6.

A length of 1024 bits was used for all binary fingerprints listed above, but for comparison a longer length of 16384 bits was used for a number of fingerprints (as in the original study). This longer version is indicated by the prefix “L”: LAvalon, LECFP6, LECFP4, LFCFP6 and LFCFP4. The Tanimoto coefficient was used to measure similarity for all binary fingerprints, while the Dice coefficient was used for count vectors.

### Dataset of similar structures

The set of all IC_50_, K_i_ and EC_50_ assays in ChEMBL 20 was used as the source for activity data. Data marked by ChEMBL as duplicates from earlier publications were discarded as these may be reference compounds with a structure distinct from the rest of the assay. The dataset was restricted to assays containing from 8 to 25 (inclusive) unique molecules. Smaller assays were found to contain more dissimilar molecules, and the value of 8 was chosen as a trade-off between retaining as much data as possible and reducing the number of dissimilar molecules retained. The upper value of 25 was chosen conservatively to limit the chance that an assay described more than one scaffold series or was selected from a HTS campaign.

ChEMBL provides ‘parent structures’ for structures in the database—this is the neutral form with any salt removed. These parent structures were used in preference to the original structure (where they differed). Furthermore, stereoisomers were normalised to the same parent compound. While in theory it would be interesting to consider differences in stereochemistry in the context of structural similarity, the presence of erroneous stereoisomers due to unspecified stereocentres meant that normalisation was required. In any case, none of the 2D fingerprints investigated in the current study are sensitive to stereochemistry.

In order to reduce the incidence of dissimilar structures in an assay, molecules were excluded from the dataset if they were members of any of the following sets. Promiscuous molecules were identified as those present in 5 or more papers in the entire set of IC_50_, K_i_ and EC_50_ data. Molecules with SMILES strings present in Wikipedia were identified using data provided by Ertl et al. [30, 31]. Finally, molecules marked by ChEMBL as having International Nonproprietary Names (INNs) were also excluded. Note that the stereo-normalised parent forms were used in each of these steps.

### Multi-assay benchmark

The multi-assay benchmark was created from the dataset of similar structures as follows. The dataset was first converted to a graph of edges connecting molecules in the same assay: inner edges connect molecules that are both in at least two papers while outer edges connect molecules where at least one is in at least two papers.

To create the first series in the benchmark, the first molecule in the dataset that was part of an outer edge was chosen. From this starting point a breadth-first search was made through the graph to create all series consisting of an outer edge, connected to an inner edge, connected to another inner edge, and finally to another outer edge. Next this set of series was filtered to ensure that no edge appeared in the same paper as another edge. To promote a diverse dataset, it was further filtered to remove any series where either end of the first edge was already present in a benchmark series as part of a first edge. If any series remain, a single one is chosen whose component molecules appear in the fewest number of papers, and this series is added to the benchmark. This procedure is repeated for each molecule in the dataset that is part of an outer edge, giving a complete benchmark dataset size of 3629 (at minimum, see next paragraph).

To assess statistical significance, 1000 repetitions of the benchmark dataset were generated by randomly shuffling all components of the dataset (i.e. the order of the molecules, the set of neighbours) and repeating the procedure described above. On average each repetition contained 3675 series, but as the minimum size was 3629, only the first 3629 series in each repetition were used for all analyses. To assess the degree of variation introduced by the repetitions, a pairwise comparison showed that successive repetitions had only 33 (±5) series in common, a value that rises to 321 (±17) when considering first two molecules in common and 673 (±20) for first molecules in common.

### Single-assay benchmark

The single-assay benchmark was created from the subset of the dataset of similar structures that contained IC_50_ and K_i_ data marked as binding assays. Within an assay, molecules marked as inactive were excluded as was the molecule (or molecules) having the lowest activity value. Also, any assay that did not contain 5 molecules with activities separated by at least 0.40 log units was discarded.

To create the first series in the benchmark, the first assay in the dataset was selected and an attempt was made to find a set of 5 molecules whose activities differ by at least 0.38 log units (this attempt involved iterating randomly over all possible selections of 5 molecules from the assay several thousand times). This procedure was repeated for each assay in the dataset that is in a different paper from those series already added to the benchmark, giving a complete benchmark dataset size of 4563 (at minimum).

To assess statistical significance, 1000 repetitions of the benchmark dataset were generated by randomly shuffling the order of the assays and repeating the procedure described above. On average each repetition contained 4573 series, but as the minimum size was 4563, only the first 4563 series in each repetition were used for all analyses. To assess the degree of variation introduced by the repetitions, a pairwise comparison showed that successive repetitions had only 331 (±13) series in common, a value that rises to 1383 (±24) when considering first two molecules in common and 2576 (±26) for first molecules in common.

### Evaluation of relative performance on the single- and multi-assay benchmarks

Each fingerprint was used to rank the second and subsequent members of each series by structural similarity with respect to the first member, the reference molecule. This ranking was compared to the order in the benchmark using the Spearman correlation. To evaluate the relative performance of two fingerprints according a single repetition of the benchmark, the following comparison statistic was used: the net difference between the number of series for which the correlation value for fingerprint A was larger than fingerprint B minus the number of series where it was smaller. Given the 1000 repetitions, a distribution of these net differences was obtained. For two fingerprints with similar performance, this distribution should be centred around zero, and in general the mean value of this distribution gives a measure of the comparative performance. The maximum possible value of the net difference is the size of the dataset.

Statistical significance at a confidence level of 1 % was assessed using a two-sided T-test (the ttest_1sampl method of SciPy [32]) where the null hypothesis is that the mean of the distribution is zero. As there are 378 pairwise comparisons we corrected for multiple comparisons using the Holm–Bonferroni correction [33]. For the single- and multi-assay benchmarks, only 4 and 5 comparisons respectively were not found to be statistically significant.

In order to summarise the data to form Hasse diagrams, it was necessary to identify incomparable fingerprints. For example, FCFP4 and AP are incomparable in the context of the multi-assay benchmark. Although the pairwise data shows that FCFP4 is better than AP, this is incompatible with pairwise data involving the HashTT fingerprint as AP is better than HashTT but the difference between HashTT and FCFP4 is not statistically significant. As a result, no edge is shown between FCFP4 and AP. For the multi-assay benchmark four fingerprint pairs were identified as incomparable, while none were identified as incomparable on the single-assay benchmark.

### Re-analysis of the Riniker–Landrum benchmark

The Riniker and Landrum benchmarking platform [9] is a ligand-based virtual screen against 88 protein targets. It is the union of three distinct datasets: 50 targets from ChEMBL 14, 21 from the Directory of Useful Decoys [34] (DUD) and 17 from the Maximum Unbiased Validation (MUV) dataset [35]. For each protein target, at least 30 actives were present. 50 repetitions of each screening experiment were carried out where 5 actives were randomly selected as the query and 20 % of the decoys were held back while the remaining actives and decoys were ranked based on maximum similarity to the query molecules (MAX fusion). The evaluation methods used in the original study included the area under the ROC curve (AUC), and a variety of early recognition methods. We focus here on results for BEDROC(20) [36] as this is the early recognition method for which the results are listed in Table 1 of the original study.

**Table 1:** Key to fingerprint abbreviations used

Abbreviation	Fingerprint description	Class
AP	Atom pair	Path-based
Avalon	Developed for substructure screen-out when searching	Substructure keys
ECFC0	Count vector form of ECFP0	Circular
ECFC2	Count vector form of ECFP2	Circular
ECFC4	Count vector form of ECFP4	Circular
ECFC6	Count vector form of ECFP6	Circular
ECFP0	Extended-connectivity fingerprint of diameter 0	Circular
ECFP2	Extended-connectivity fingerprint of diameter 2	Circular
ECFP4	Extended-connectivity fingerprint of diameter 4	Circular
ECFP6	Extended-connectivity fingerprint of diameter 6	Circular
FCFC2	Count vector form of FCFP2	Circular
FCFC4	Count vector form of FCFP4	Circular
FCFC6	Count vector form of FCFP6	Circular
FCFP2	Feature-class fingerprint of diameter 2	Circular
FCFP4	Feature-class fingerprint of diameter 4	Circular
FCFP6	Feature-class fingerprint of diameter 6	Circular
HashAP	Bit vector form of AP	Path-based
HashTT	Bit vector form of TT	Path-based
LAvalon	16384-bit form of Avalon	Substructure keys
LECFP4	16384-bit form of ECFP4	Circular
LECFP6	16384-bit form of ECFP6	Circular
LFCFP4	16384-bit form of FCFP4	Circular
LFCFP6	16384-bit form of FCFP6	Circular
MACCS	Molecular ACCess System structural keys	Substructure keys
RDK5	Encodes paths of maximum length 5	Path-based
RDK6	Encodes paths of maximum length 6	Path-based
RDK7	Encodes paths of maximum length 7	Path-based
TT	Topological torsion fingerprint	Path-based

See “Methods” section for associated references

The datasets and software used to produce the results were included with the paper and also deposited in a GitHub repository [24]. We reran the benchmark procedure using the code in the repository and version 2015.09.2 of the RDKit cheminformatics toolkit. The original analysis of the benchmark results ranked the fingerprints for each repetition using a specific evaluation method [for example, BEDROC(20)], took the mean of these ranks over the 50 repetitions and then the mean over all of the proteins. The statistical significance of the pairwise differences was assessed using a bootstrapping procedure that sampled with replacement the ranks from each repetition. Sheridan [37] has also advocated the use of mean ranks instead of mean scores, as the latter are highly influenced by those proteins with a large range of scores. However the use of the mean rank is itself problematic as the pairwise similarity of two methods can be altered (and even inverted) by adding additional methods to an evaluation.

We avoided these problems by measuring the relative performance of two fingerprints using the same method described above for the new benchmarks. Using the data from a single repetition of the Riniker–Landrum benchmark, the following comparison value was used for each pair of fingerprints A and B: the net difference between the number of protein targets for which the evaluation method for fingerprint A was better than fingerprint B minus the number of targets where it was worse. Given the 50 repetitions, a distribution of this net difference was obtained. For two fingerprints with similar performance, this distribution should be centred around zero, and in general the mean value of this distribution gives a measure of the comparative performance. The maximum possible value of the net difference is the size of the dataset, 88.

The remainder of the analysis is the same as that described above for the single-assay and multi-assay benchmarks. Of the 378 pairwise comparisons, 35 were not found to be statistically significant at the 1 % level. No fingerprint pairs were found to be incomparable. Compared to the analysis in the original paper, this approach has greater statistical power; for the original analysis, 46 of the 91 pairwise comparisons (of 14 fingerprints) were not statistically significant at the 5 % level. Qualitatively the results are largely in agreement, although a comparison with Table 1 in the original study does highlight some differences; for example, RDK5 performs worse in our analysis, and the order of LAvalon and Avalon is reversed.

## Results and discussion

### Identifying structurally similar molecules from ChEMBL assays

Both of the new benchmarks use co-appearance in the same ChEMBL assay as an indication that two molecules are structurally similar (according to the definition in the Introduction). However, the naïve assumption that all molecules in the same ChEMBL assay are structurally similar is of course not true. Molecules already known to modulate the activity (e.g. inhibitors or agonists) are sometimes included for comparison; known inactives may be present as internal controls; assays may include several different chemotypes or scaffolds; indeed, the molecules in an assay may bear no structural similarity beyond hitting a particular target (e.g. the result of a HTS campaign). While it is tempting to use a similarity measure to filter out structures that are dissimilar to the majority of an assay, this must be avoided as it would bias the results.

**Fig. 2:**
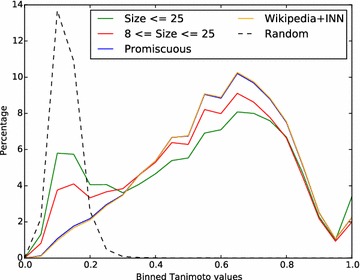
Histogram showing the effect of successive filters on the pairwise similarity of structures in the same assay. Pairwise similarity was measured using the LECFP4 fingerprint for pairs of structures from each assay in the dataset and a histogram generated using a bin width of 0.05. The initial data (*green*) was for assays containing up to 25 structures. Successive filters were then applied to restrict the data to those assays of size 8 or greater, to remove promiscuous molecules, and to remove molecules found in Wikipedia or with INNs. For comparison, the pairwise similarity of randomly chosen molecules from the entire dataset is shown as the *dashed line*. Histograms were normalised to 100 % over all bins, except for the histogram for the random data which was scaled to 30 %

Instead we have used a series of filters that indirectly remove data that has a high probability of being dissimilar (see “Methods” section). To begin with, the dataset is restricted to assays containing between 8 and 25 molecules (inclusive). Then molecules are removed if they appear in more than four papers (promiscuous molecules), if they are present in Wikipedia or if they have an INN. The effect of each successive filter on the within-assay pairwise similarity is shown in Fig. 2. With each filter, the peak at a Tanimoto value of 0.1 is reduced, an indication that structures dissimilar to the majority of the assay have been removed. Although the effect of eliminating Wikipedia structures may be small compared to earlier filters, visual inspection of benchmark series containing Wikipedia structures indicated that in about half of the cases the Wikipedia structure was not structurally similar to at least one of its immediate neighbours in the series. A similar inspection for the few remaining structures with INNs did not indicate that retaining them would cause any problem but it seemed a reasonable precaution to also remove those.

Naturally the procedure described here also removes a large amount of acceptable data in the context of many assays but this is unavoidable. It also will not eliminate all dissimilar structures since it is only able to identify such structures indirectly. Despite this, the procedure described was quite successful as illustrated by the reduction in the size of the peak at 0.1 Tanimoto in Fig. 2, and this is supported by inspection of the series generated for each benchmark.

### Ordering molecules by structural similarity relative to a reference

In order to test a fingerprint’s ability to order molecules by structural similarity, we created two distinct benchmark datasets of thousands of series of 5 molecules, each series containing a reference molecule and an ordered arrangement of 4 molecules.

**Fig. 3:**
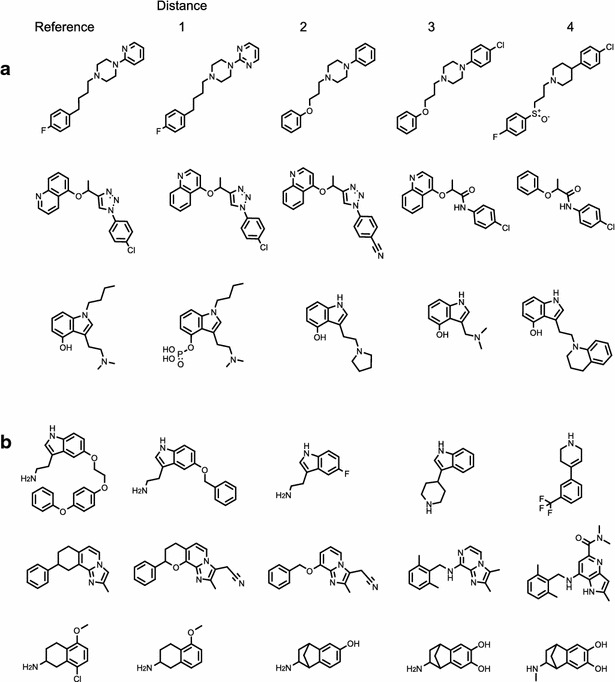
Examples of series from **a** the single-assay benchmark, **b** the multi-assay benchmark

The single-assay benchmark consists of 4563 series each containing five molecules from the same ChEMBL assay where none of the five molecules is within 0.38 activity units of another. The members of each series are ordered by their activity with respect to the most active (the reference molecule). Examples from three different publications are shown in Fig. 3a [38–40]. The basis for the use of these data as a benchmark for structural similarity is our assumption that structural similarity decreases relative to the reference molecule as one moves across the series to lower activity. While this procedure will not work for an arbitrary set of molecules active against a particular target (due to the presence of different scaffolds, binding modes and other confounding effects), it works in this case due to the constraint of our definition of similarity.

The multi-assay benchmark consists of 4563 series containing five molecules where the first two are from the same ChEMBL assay, the third comes from a different paper but co-occurs with the second, the fourth from a different paper but co-occurs with the third, and similarly the fifth. Three examples are shown in Fig. 3b each of which is based on structures from four publications [41–52]. The basis for the use of these data as a benchmark for structural similarity is the assumption that structural similarity decreases relative to a reference molecule as one moves from one paper to another through co-occurring molecules, given the size of chemical space and the nature of a random walk.

The assumptions listed above will not always be true, but this is not a problem so long as any other effects randomly order the molecules. Of course, such randomly-ordered series will add to the noise and set an upper bound on performance.

**Fig. 4:**
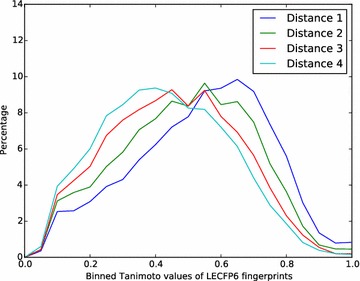
Histogram showing the structural similarity of structures in the single-assay benchmark with respect to their corresponding reference molecules. Similarity was measured with the LECFP6 fingerprint and a histogram created using bins of width 0.05. Histograms were normalised to 100 % over all bins. The data used here is taken from all 1000 repetitions of the benchmark

**Fig. 5:**
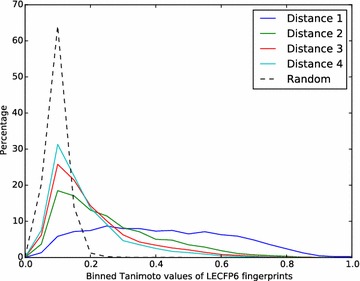
Histogram showing the structural similarity of structures in the multi-assay benchmark with respect to their corresponding reference molecules. Similarity was measured with the LECFP6 fingerprint and a histogram created using bins of width 0.05. Histograms were normalised to 100 % over all bins. The data used here is taken from all 1000 repetitions of the benchmark

Figures 4 and 5 shows the distributions of Tanimoto values for the LECFP6 fingerprints of the four molecules in the series relative to the reference molecule. In both cases there is a clear shift to lower similarity values on moving across the series, which supports our assumption. This shift is much more marked for the multi-assay benchmark compared to the single-assay benchmark, which is expected given that the structures in the single-assay benchmark are all from the same paper (and assay). As the structural differences in the multi-assay benchmark are greater, it should present an easier task for fingerprints and so the performance is likely to be better.

### Benchmarking fingerprint performance

Each fingerprint was assessed for its ability to reproduce the series order for each series in the datasets, 4563 series in the case of the single-assay benchmark and 3629 for the multi-assay benchmark. That is, can the similarity measure correctly order four query molecules with respect to a reference molecule?

**Fig. 6:**
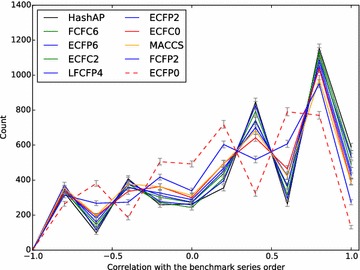
Histogram of the performance for a subset of the fingerprints on the single-assay benchmark. The *graph* depicts the number of times a fingerprint had a particular correlation with the benchmark series order. The order of fingerprints in the legend matches the counts at correlation 1.0. The *colours* used correspond to those used by Riniker and Landrum [9] in their Fig. 5. The correlation values used in the graph cover all the possible rank correlations between two ordered series of length 4; intermediate values (due to ties) were rounded to the next lowest value for positive values and the next largest value for negative values

**Fig. 7:**
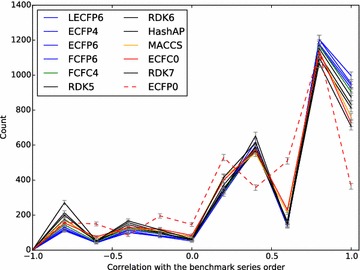
Histogram of the performance for a subset of the fingerprints on the multi-assay benchmark. See caption for Fig. 6 for more details

Figures 6 and 7 give an overview of the average performance for a subset of the fingerprints. If we consider the multi-assay benchmark first (Fig. 7), one of the best performing fingerprints (LECFP4) can reproduce or almost reproduce (off-by-one) the original series order in 2201 of the 3629 cases (61 %). This compares to the baseline ECFP0 fingerprint which can do so in only 1498 of the cases (41 %). The single-assay benchmark presents a more challenging set of series to be ordered; one of the best performing fingerprints (HashAP) only gets the answer close to correct for 1744 of the 4563 cases (38 %). The corresponding value for the ECFP0 fingerprint is 942 (20 %).

While Figs. 6 and 7 gives an overview of the average performance of different fingerprints, a method with more statistical power was used to calculate the relative performance of each fingerprint. If one considers a single repetition from either of the benchmarks, there are several thousand independent test cases. For each one, it is possible to rank the various fingerprints by how well they perform. Using these ranks, for each pair of fingerprints A and B one can calculate the net difference between the number of times A performed better than B minus the number of times it performed worse. The distributions of these net differences over 1000 repetitions give a mean net difference for each pair as well as the ability to test statistical significance. A similar method was used to assess fingerprint performance for the Riniker–Landrum virtual screening benchmark, for which 50 repetitions were present.

**Fig. 8:**
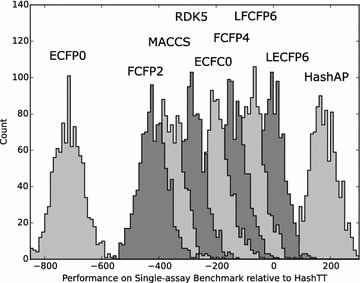
Histograms showing the relative performance of a subset of the fingerprints relative to the HashTT fingerprint in the single-assay benchmark. Relative performance was measured for each of the 1000 repetitions by counting the number of times a particular fingerprint had higher correlation with the benchmark series compared to the HashTT fingerprint and subtracting the number of times it had lower correlation. The difference between the performance of the LECFP6 and HashTT fingerprints was not found to be statistically significant. All others fingerprints shown were either better (those to the *right*) or worse (those to the *left*) than HashTT. A bin width of 10 was used

**Fig. 9:**
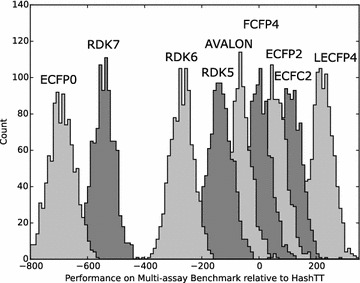
Histograms showing the relative performance of a subset of the fingerprints relative to the HashTT fingerprint in the multi-assay benchmark. Relative performance was measured for each of the 1000 repetitions by counting the number of times a particular fingerprint had higher correlation with the benchmark series compared to the HashTT fingerprint and subtracting the number of times it had lower correlation. The difference between the performance of the FCFP4 and HashTT fingerprints was not found to be statistically significant. All others fingerprints shown were either better (those to the *right*) or worse (those to the *left*) than HashTT. A bin width of 10 was used

**Fig. 10:**
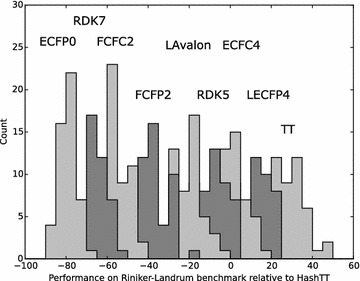
Histograms showing the relative performance of a subset of the fingerprints relative to the HashTT fingerprint in the Riniker–Landrum benchmark. Relative performance was measured for each of the 50 repetitions by counting the number of times a particular fingerprint had a higher BEDROC(20) value compared to the HashTT fingerprint and subtracting the number of times it had a lower value. The difference between the performance of the ECFC4 and HashTT fingerprints was not found to be statistically significant. All others fingerprints shown were either better (those to the *right*) or worse (those to the *left*) than HashTT. A bin width of 5 was used

 Figures 8, 9 and 10 show a subset of these distributions versus the HashTT fingerprint for each of the three benchmarks. The mean values of the histograms partition the fingerprints into sets that are better than HashTT, worse than HashTT, and those where there is no significant difference. HashTT was chosen here as an example because its average performance across all of the benchmarks illustrates this partitioning. These mean values of the net difference are the primary result of the benchmarks and the complete matrices are included in the Additional file 1 as tab-separated files. The Additional file 2 includes 2D embeddings of the distance matrix created from the absolute values of the net differences; this gives an overview of the relative magnitude of the differences between fingerprints.

### Which fingerprint best corresponds to the literature-based measures of structural similarity?

As a measure of the relative performance of two fingerprints A and B on a benchmark, we used the net difference between the number of times A performed better than B minus the number of times it performed worse. Combined with multiple repetitions, this enabled us to make statistically significant comparisons between the majority of the fingerprints tested.

**Fig. 11:**
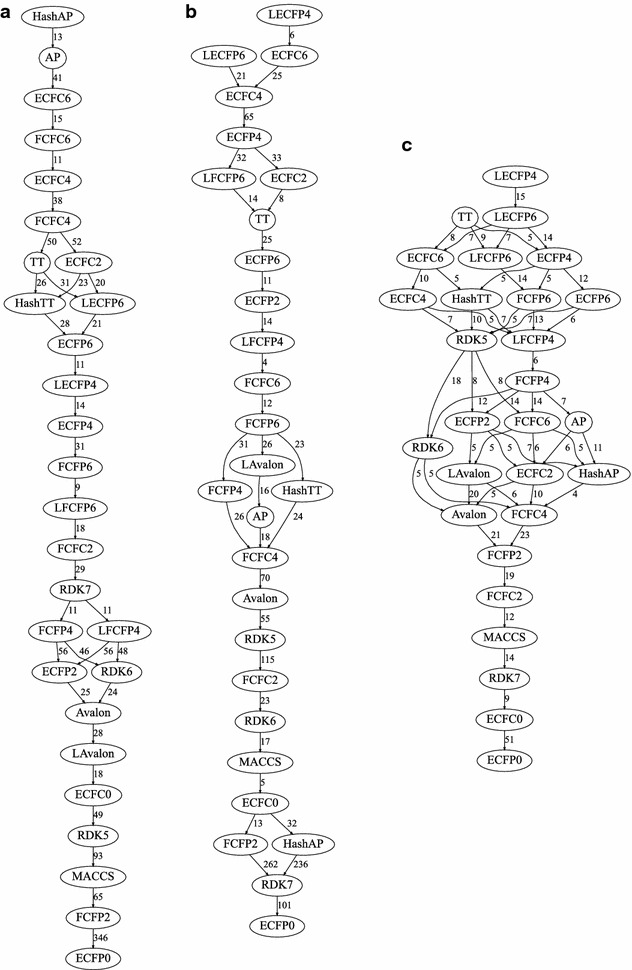
Directed graphs summarising the relative performance on **a** the single-assay benchmark, **b** the multi-assay benchmark and **c** the Riniker–Landrum benchmark. All fingerprints higher in the graph are better than those below if a path between them exists. The *numbers* indicate the net difference (number of times better minus number of times worse) for neighbouring fingerprints. Note that the scales are different as the upper values are bounded by the dataset size: 3629 for **a**, 4563 for **b**, and 88 for **c**. Note that the net differences may not obey the triangle inequality. To find out the net difference for a non-neighbouring pair, see the tables in the Additional file 1

Figure 11a and b summarise the relative performance of the fingerprints based on the complete set of net differences for both the single- and multi-assay benchmark. Figure 11c shows the corresponding information for Riniker–Landrum benchmark. It should be noted first of all that the layout of this graph representation (strictly speaking, a Hasse diagram) does not make clear the magnitude of the distances between fingerprints. For example, while HashAP is the best performing fingerprint in the single-assay benchmark, its distance to TT (six positions below it in the graph) is 149 which corresponds to just 3.3 % of the dataset. On the other hand, given that a certain proportion of the dataset will contain series where the structures are incorrectly ordered (for example, consider that ECFP0 has on average a better correlation with the series order than LECFP4 on ~900 of the 3629 series in the multi-assay benchmark), a better way to calibrate this value may be to consider it as 16 % of the distance between the best and worst fingerprints.

Looking at Fig. 11 it is clear that the overall performance of the fingerprints is only moderately similar between the single- and multi-assay benchmarks, but that there is quite good agreement between the results from the virtual screen and the multi-assay benchmark. These similarities can be quantified by calculating a correlation value between the net difference matrices based on the concordance of pairs, that is, whether A and B are relatively ordered the same in both matrices. The correlation value (Kendall tau) between the multi-assay and virtual screen benchmarks is 0.68 (0.76 if only using statistically significant values). The corresponding value for the single-assay benchmark versus the multi-assay and virtual screen are 0.39 and 0.35 respectively (0.40 and 0.39 for significant values).

Considering first the results from the multi-assay and virtual screening benchmarks, the LECFP4 and LECFP6 fingerprints are consistently among the best tested. In fact, among the top fingerprints only the topological torsion (TT) fingerprint is not an extended-connectivity or feature-class fingerprint. At the other end of the scale, the ECFP0, RDK7, ECFC0 and MACCS fingerprints are the poorest performers.

The single-assay benchmark requires the fingerprints to correctly order very similar structures, many of which are matched pairs or contain minor modifications to scaffolds. Compared to the multi-assay benchmark, the most notable difference in the results depicted in Fig. 11a is that the atom pair fingerprints, AP and HashAP, move from being average and poor (respectively) to being the best performers. The atom pair fingerprint [27] encodes all pairs of atoms along with the shortest distance between them. Note that, unlike a Daylight-type fingerprint such as RDK5, the path itself is not encoded and the environment of an atom is only encoded to the extent of counting the number of heavy atoms attached. This means that structural modifications that only change a few atoms (e.g. changing a carbon to a nitrogen, an R-group replacement, or moving an R-group around a ring) will only have a small effect on the value.

Another notable difference between the benchmarks is that the count vector forms of the four circular fingerprints with diameter 4 and 6 improved the results for the single-assay benchmark, but were uniformly worse for the multi-assay and Riniker–Landrum benchmarks (except for a single instance where the value not was significantly different). The key difference between the xCFC (count vector) and xCFP (bit vector) fingerprints is that where the same structural feature is observed more than once the count vectors record the number of observations while the bit vector forms just record its presence. As the count vector is more information-rich, one would expect the corresponding similarity measurements to always perform better. Looking at the results for the multi-assay benchmark, while the results for LECFP6 are not (statistically) significantly different than those for ECFC6, the net difference for LECFP4 versus ECFC4 is 23, that for LFCFP4/FCFC4 is 71 and that for LFCFP6/LFCFC6 is also 71. These differences are unlikely to be due to collisions as the count vector as implemented in RDKit is a sparse vector of length 4294967296 bits. Having inspected a number of series where LECFP4 outperforms ECFC4, we are still unsure why this difference is observed. It must be that penalising the absence of a second (or third, etc.) feature to the same extent as penalising the complete absence of a feature yields a worse similarity measure. In other words, that there is a large gain in similarity in having a bit (or equivalently a count of 1) in common between two fingerprints, but that increasing the count in common to 2 does not increase the similarity to the same extent.

This still leaves the question of why the performance of the count vector forms is better on the single-assay benchmark. The explanation may be a molecular weight effect. If we include the absolute difference in the molecular weight as a method of ordering the benchmark series, in the multi-assay benchmark it had a poorer net difference than ECFP0 on 845 of the repetitions. In contrast, it slightly outperforms ECFP0 in the single-assay benchmark (by a net difference of 33). As the count vector forms of the circular fingerprints are more sensitive than the bit-vector forms to similarity in molecular weight, this may explain their improved performance in the single-assay benchmark.

It is to be expected that a shorter fingerprint length will introduce collisions thus adding noise to the calculation of similarity with a consequent loss of performance. This was observed by Sastry et al. [18] for Daylight fingerprints with 1024 bits. To investigate this, Riniker and Landrum included five pairs of fingerprints as both the default length (1024 bits) and a long form (16,384 bits). In their analysis, no considerable improvement in performance was observed but when re-analysed we find that in each case the long form performed better. This was also the case for the multi-assay benchmark; the net difference on the multi-assay benchmark for the LAvalon fingerprint versus the Avalon fingerprint was 132, while that for LECFP6/ECFP6 was 172, LECFP4/ECFP4 was 99, LFCFP6/FCFP6 was 96 and LFCFP4/FCFP4 was 57. As a larger number of bit collisions would be observed for the xCFP6 fingerprints versus xCFP4, the larger performance improvements found for the ECFP6 and FCFP6 fingerprints are expected. In contrast, the single-assay benchmark did not show a clear trend; the Avalon and FCFP6 fingerprints were better than their long forms by 28 and 9 respectively, while the LECFP6 and LECFP4 fingerprints were better than their short forms by 21 and 14 respectively (the LFCFP4/FCFP4 difference was not statistically significant).

## Conclusions

To our knowledge, the concepts behind both of the new benchmarks have not previously been explored. They present a number of advantages over previous benchmarks:

1. The new benchmarks achieve a degree of separation of different fingerprints that is an order of magnitude greater than a virtual screening benchmark. This is partly due to their size; each of several thousand series has a vote on which of two fingerprints is better, compared to fewer than 100 for a large-scale virtual screen.
2. The new benchmarks are not prone to the problems associated with using the mean score or the mean rank to evaluate methods, problems which have reduced the statistical power of previous studies. Strictly speaking these problems can be avoided even for virtual screens, as we show in our re-analysis of the Riniker–Landrum study, but they are endemic in the field. Instead of taking the means and then comparing them, our approach is to do the pairwise comparisons first for each target (or series) and then collate the results.
3. The new benchmarks are intended to measure ability to rank structures by similarity, whereas a benchmark for virtual screening only measures this indirectly. For example, the use of multiple query structures in a virtual screen (as in the Riniker and Landrum study) is considered good practice as it improves recall [53, 54]. However, this is a confounding factor when considering the ability to measure structural similarity. Good performance for one fingerprint might be due to structural similarity to one of the query structures, but for a different fingerprint good performance might be due to similarity to another.
4. The issue of which metric to use to evaluate a virtual screen has made it difficult to compare and indeed assess studies, especially as the majority have not made their results available as part of the publication or provided a way to reproduce them. A particular problem is the continued use of metrics related to the average rank of the actives and that, as a result, cannot even distinguish between trivial examples of methods with good versus poor performance [36]. For the single- and multi-assay benchmarks, the evaluation metric is the rank correlation.
5. Compared to a virtual screen, which assumes that actives are more similar to each other than to inactives, the new benchmarks are based upon different assumptions: namely, that within an assay molecules with similar activity tend to have similar structures, and that structural similarity decreases as one moves from one paper to another through molecules in common between both. Furthermore, the degree of similarity varies in each case. Given these differences, one can compare the results of the new benchmarks with a virtual screen to determine to what extent structural similarity is the same in different contexts.

While it is easy to assert that fingerprint performance depends on the particular context, it is nonetheless the case that certain fingerprints are more likely than others to perform well in general.

Here we provide conclusive evidence that extended-connectivity fingerprints of diameter 4 and 6 are among the best performing fingerprints whether separating actives from decoys in a virtual screen or ranking diverse structures by similarity. The topological torsion fingerprint is also found to perform well at these tasks. For the specific case of ranking very similar structures (for example, close analogues), the atom pair fingerprint outperforms the others tested, with the count versions of the extended-connectivity fingerprints also performing well. Fingerprints to avoid when measuring similarity include Daylight-type path-based fingerprints and MACCS keys. It also appears that using fingerprints longer than 1024 bits is worthwhile due to the improved performance. Finally, given the sensitivity of the multi-assay benchmark and the degree of agreement with the BEDROC(20) results for the virtual screen, we believe that the multi-assay benchmark will prove useful for the development of improved fingerprints for virtual screening.

## Supporting information

 The datasets and Python scripts to reproduce the results are available from https://github.com/nextmovesoftware/similaritybenchmark.

## Additional files

10.1186/s13321-016-0148-0 The matrices of net differences for each of the benchmarks.
10.1186/s13321-016-0148-0 2D visualisations of the net difference matrices.

## References

[1] Johnson MA, Maggiora GM (1990). Concepts and applications of molecular similarity.

[2] Maggiora GM (2006). On outliers and activity cliffs—why QSAR often disappoints. J Chem Inf Model.

[3] Willett P (2014). The calculation of molecular structural similarity: principles and practice. Mol Inform.

[4] Muegge I, Mukherjee P (2016). An overview of molecular fingerprint similarity search in virtual screening. Expert Opin Drug Discov.

[5] Stumpfe D, Bajorath J (2011). Similarity searching. Wiley Interdiscip Rev Comput Mol Sci.

[6] Cereto-Massagué A, Ojeda MJ, Valls C (2015). Molecular fingerprint similarity search in virtual screening. Methods.

[7] McGaughey GB, Sheridan RP, Bayly CI (2007). Comparison of topological, shape, and docking methods in virtual screening. J Chem Inf Model.

[8] Venkatraman V, Pérez-Nueno VI, Mavridis L, Ritchie DW (2010). Comprehensive comparison of ligand-based virtual screening tools against the DUD data set reveals limitations of current 3D methods. J Chem Inf Model.

[9] Riniker S, Landrum GA (2013). Open-source platform to benchmark fingerprints for ligand-based virtual screening. J Cheminf.

[10] Tiikkainen P, Markt P, Wolber G (2009). Critical comparison of virtual screening methods against the MUV data set. J Chem Inf Model.

[11] Heikamp K, Bajorath J (2011). Large-scale similarity search profiling of ChEMBL compound data sets. J Chem Inf Model.

[12] Patterson DE, Cramer RD, Ferguson AM (1996). Neighborhood behavior: a useful concept for validation of “molecular diversity” descriptors. J Med Chem.

[13] Horvath D, Jeandenans C (2003). Neighborhood behavior of in silico structural spaces with respect to in vitro activity spaces—a novel understanding of the molecular similarity principle in the context of multiple receptor binding profiles. J Chem Inf Comput Sci.

[14] Papadatos G, Cooper AWJ, Kadirkamanathan V (2009). Analysis of neighborhood behavior in lead optimization and array design. J Chem Inf Model.

[15] Steffen A, Kogej T, Tyrchan C, Engkvist O (2009). Comparison of molecular fingerprint methods on the basis of biological profile data. J Chem Inf Model.

[16] Hert J, Willett P, Wilton DJ (2004). Comparison of topological descriptors for similarity-based virtual screening using multiple bioactive reference structures. Org Biomol Chem.

[17] Bender A, Jenkins JL, Scheiber J (2009). How similar are similarity searching methods? a principal component analysis of molecular descriptor space. J Chem Inf Model.

[18] Sastry M, Lowrie JF, Dixon SL, Sherman W (2010). Large-scale systematic analysis of 2D fingerprint methods and parameters to improve virtual screening enrichments. J Chem Inf Model.

[19] Briem H, Lessel UF (2000). In vitro and in silico affinity fingerprints: finding similarities beyond structural classes. Perspect Drug Discov Des.

[20] Duan J, Dixon SL, Lowrie JF, Sherman W (2010). Analysis and comparison of 2D fingerprints: insights into database screening performance using eight fingerprint methods. J Mol Graph Model.

[21] Franco P, Porta N, Holliday JD, Willett P (2014). The use of 2D fingerprint methods to support the assessment of structural similarity in orphan drug legislation. J Cheminf.

[22] Maggiora G, Vogt M, Stumpfe D, Bajorath J (2014). Molecular similarity in medicinal chemistry. J Med Chem.

[23] Bento AP, Gaulton A, Hersey A (2014). The ChEMBL bioactivity database: an update. Nucleic Acids Res.

[24] Riniker S, Landrum G (2016) Code repository for benchmarking platform. https://github.com/rdkit/benchmarking_platform. Accessed 15 Jan 2016

[25] RDKit (2016) Cheminformatics and machine learning software. http://rdkit.org/. Accessed 15 Jan 2016

[26] Nilakantan R, Bauman N, Dixon JS, Venkataraghavan R (1987). Topological torsion: a new molecular descriptor for SAR applications. Comparison with other descriptors. J Chem Inf Model.

[27] Carhart RE, Smith DH, Venkataraghavan R (1985). Atom pairs as molecular features in structure-activity studies: definition and applications. J Chem Inf Comput Sci.

[28] Gedeck P, Rohde B, Bartels C (2006). QSAR—how good is it in practice? comparison of descriptor sets on an unbiased cross section of corporate data sets. J Chem Inf Model.

[29] Rogers D, Hahn M (2010). Extended-connectivity fingerprints. J Chem Inf Model.

[30] Ertl P, Patiny L, Sander T (2015). Wikipedia chemical structure explorer: substructure and similarity searching of molecules from Wikipedia. J Cheminf.

[31] Wikipedia structure search (2016) http://www.cheminfo.org/wikipedia/. Accessed 15 Jan 2016

[32] Jones E, Oliphant T, Peterson P (2001). others.

[33] Holm S (1979). A simple sequentially rejective multiple test procedure. Scand J Stat.

[34] Huang N, Shoichet BK, Irwin JJ (2006). Benchmarking sets for molecular docking. J Med Chem.

[35] Rohrer SG, Baumann K (2009). Maximum unbiased validation (MUV) data sets for virtual screening based on PubChem bioactivity data. J Chem Inf Model.

[36] Truchon J-F, Bayly CI (2007). Evaluating virtual screening methods: good and bad metrics for the “early recognition” problem. J Chem Inf Model.

[37] Sheridan RP (2008). Alternative global goodness metrics and sensitivity analysis: heuristics to check the robustness of conclusions from studies comparing virtual screening methods. J Chem Inf Model.

[38] Peprah K, Zhu XY, Eyunni SVK (2012). Multi-receptor drug design: Haloperidol as a scaffold for the design and synthesis of atypical antipsychotic agents. Bioorg Med Chem.

[39] Maurya SK, Gollapalli DR, Kirubakaran S (2009). Triazole inhibitors of *Cryptosporidium parvum* inosine 5′-monophosphate dehydrogenase. J Med Chem.

[40] Sard H, Kumaran G, Morency C (2005). SAR of psilocybin analogs: discovery of a selective 5-HT2C agonist. Bioorg Med Chem Lett.

[41] Meng H, Liu Y, Zhai Y, Lai L (2013). Optimization of 5-hydroxytryptamines as dual function inhibitors targeting phospholipase A2 and leukotriene A4 hydrolase. Eur J Med Chem.

[42] DeFalco J, Steiger D, Dourado M (2010). 5-Benzyloxytryptamine as an antagonist of TRPM8. Bioorg Med Chem Lett.

[43] Matzen L, van Amsterdam C, Rautenberg W (2000). 5-HT reuptake inhibitors with 5-HT1B/1D antagonistic activity: a new approach toward efficient antidepressants. J Med Chem.

[44] Conway RJ, Valant C, Christopoulos A (2012). Synthesis and SAR study of 4-arylpiperidines and 4-aryl-1,2,3,6-tetrahydropyridines as 5-HT2C agonists. Bioorg Med Chem Lett.

[45] Palmer AM, Münch G, Brehm C (2008). 5-Substituted 1*H*-pyrrolo[3,2-b]pyridines as inhibitors of gastric acid secretion. Bioorg Med Chem.

[46] Palmer AM, Grobbel B, Brehm C (2007). Preparation of tetrahydroimidazo[2,1-a]isoquinolines and their use as inhibitors of gastric acid secretion. Bioorg Med Chem.

[47] Kaminski JJ, Wallmark B, Briving C, Andersson BM (1991). Antiulcer agents. 5. Inhibition of gastric H+/K+-ATPase by substituted imidazo[1,2-a]pyridines and related analogs and its implication in modeling the high affinity potassium ion binding site of the gastric proton pump enzyme. J Med Chem.

[48] Panchal T, Bailey N, Bamford M (2009). Evaluation of basic, heterocyclic ring systems as templates for use as potassium competitive acid blockers (pCABs). Bioorg Med Chem Lett.

[49] DeMarinis RM, Shah DH, Hall RF (1982). α-adrenergic agents. 2. Synthesis and α1-agonist activity of 2-aminotetralins. J Med Chem.

[50] Grunewald GL, Bartlett WJ, Reitz TJ (1986). Conformationally defined adrenergic agents. 9. Binding requirements of phenolic phenylethylamines in the benzonorbornene skeleton at the active site of phenylethanolamine N-methyltransferase. J Med Chem.

[51] Ye Q, Grunewald GL (1989). Conformationally defined adrenergic agents. 15. Conformationally restricted and conformationally defined tyramine analogs as inhibitors of phenylethanolamine *N*-methyltransferase. J Med Chem.

[52] Burn P, Crooks PA, Heatley F (1982). Synthesis and dopaminergic properties of some exo- and endo-2-aminobenzonorbornenes designed as rigid analogs of dopamine. J Med Chem.

[53] Nasr RJ, Swamidass SJ, Baldi PF (2009). Large scale study of multiple-molecule queries. J Cheminf.

[54] Whittle M, Gillet VJ, Willett P, Loesel J (2006). Analysis of data fusion methods in virtual screening: similarity and group fusion. J Chem Inf Model.

