# Supplementary material for: Comparing structural fingerprints using a literature-based similarity benchmark
Source: J Cheminform. 2016 Jul 5;8:36. doi: 10.1186/s13321-016-0148-0 (PMC4932683; doi:10.1186/s13321-016-0148-0)
Supplement: Supplementary file 2 — 10.1186/s13321-016-0148-0 2D visualisations of the net difference matrices. [file 13321_2016_148_MOESM2_ESM.docx]

# Supporting Information

## Pairwise net differences

The pairwise net differences between the 28 fingerprints are listed in tab-separated files which may be opened with (for example) Excel. Positive values indicate that the row name was better than the column name, while negative values indicate that the row name was worse than the column name. Values marked by asterisks were not found to be statistically significant.

## 2D visualisation of net difference matrices


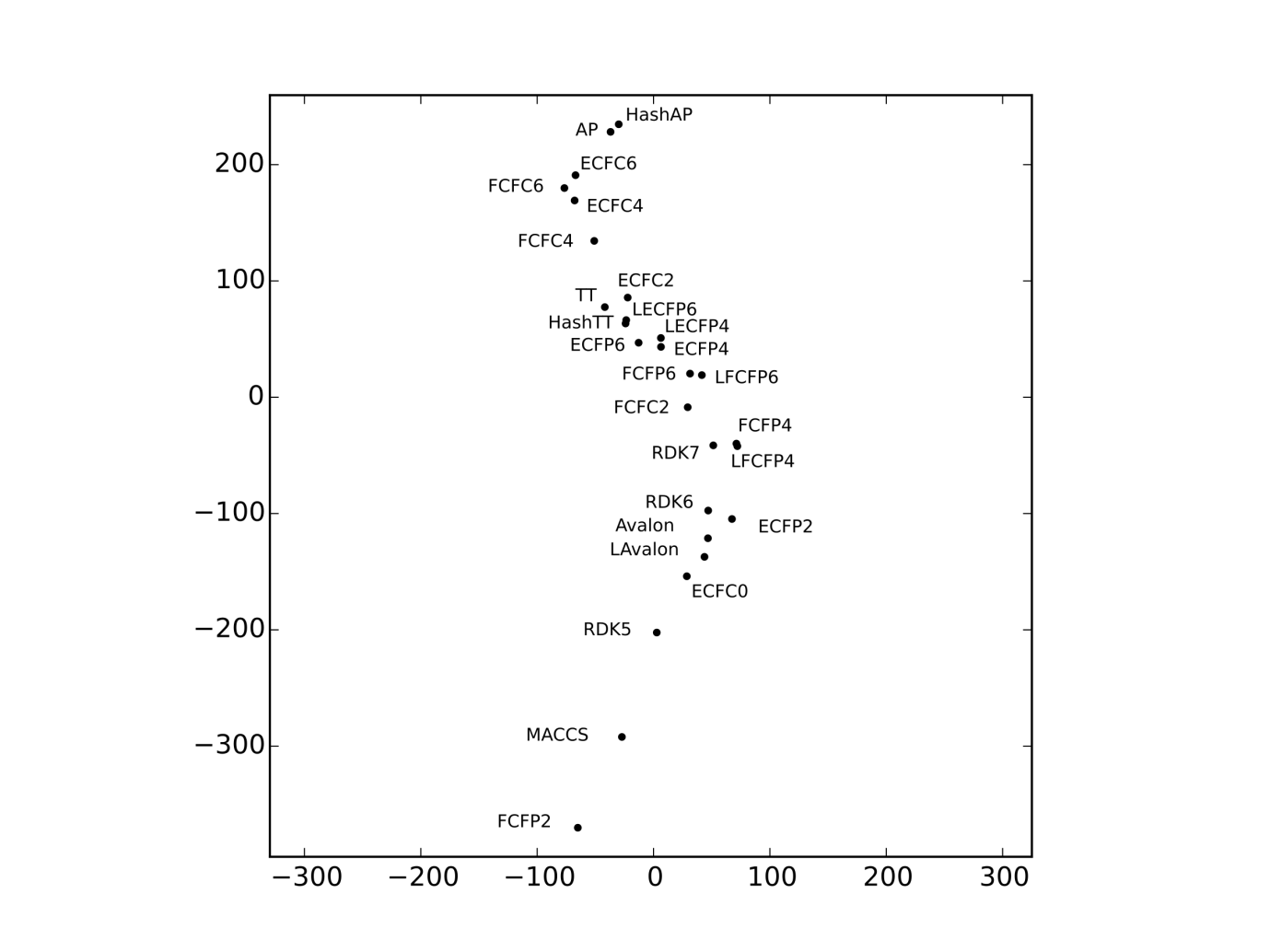


**Figure S1 – A representation of the single-assay benchmark net difference matrix in 2D, created using multi-dimensional scaling.** This provides an overview of the magnitude of the relative performances of the fingerprints. The absolute values of the net differences were used as distances for input to the multi-dimensional scaling method. To enhance clarity, the ECFP0 fingerprint has been excluded from the procedure.


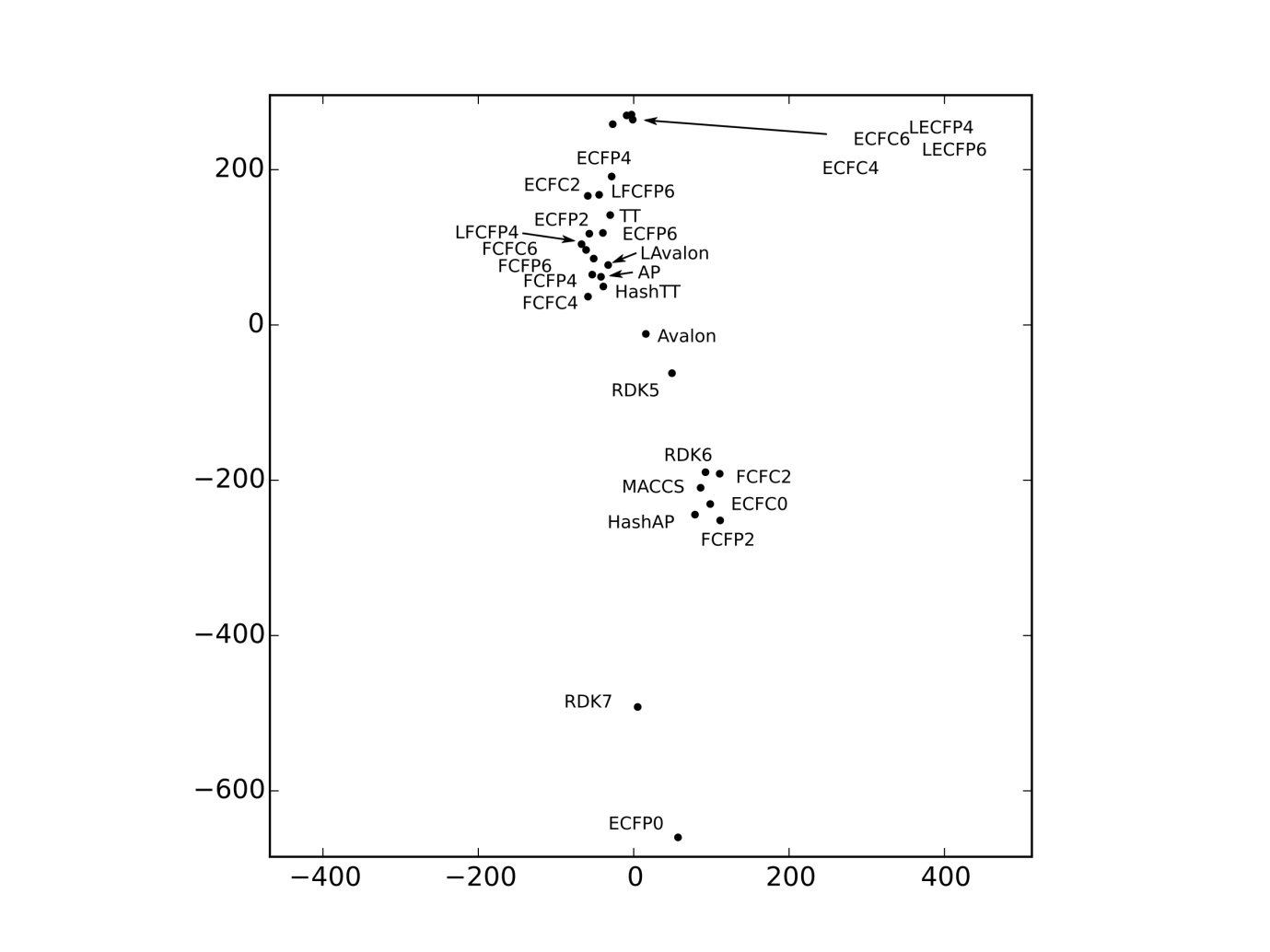


**Figure S2 – A representation of the multi-assay benchmark net difference matrix in 2D, created using multi-dimensional scaling.**


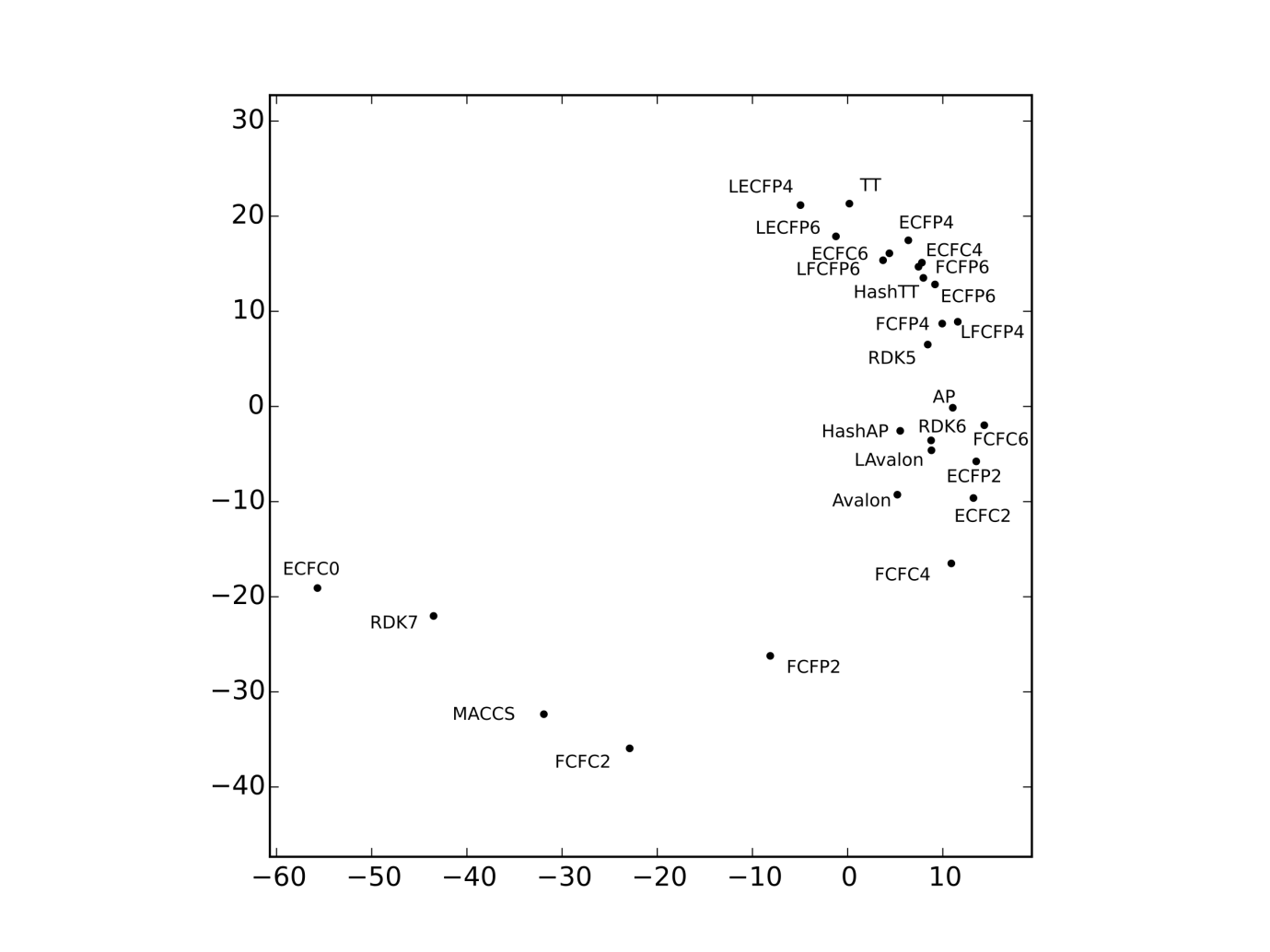


**Figure S3 – A representation of the Riniker-Landrum benchmark net difference matrix in 2D, created using multi-dimensional scaling.** To enhance clarity, the ECFP0 fingerprint has been excluded from the procedure.

## 2D visualisation of fingerprint similarity


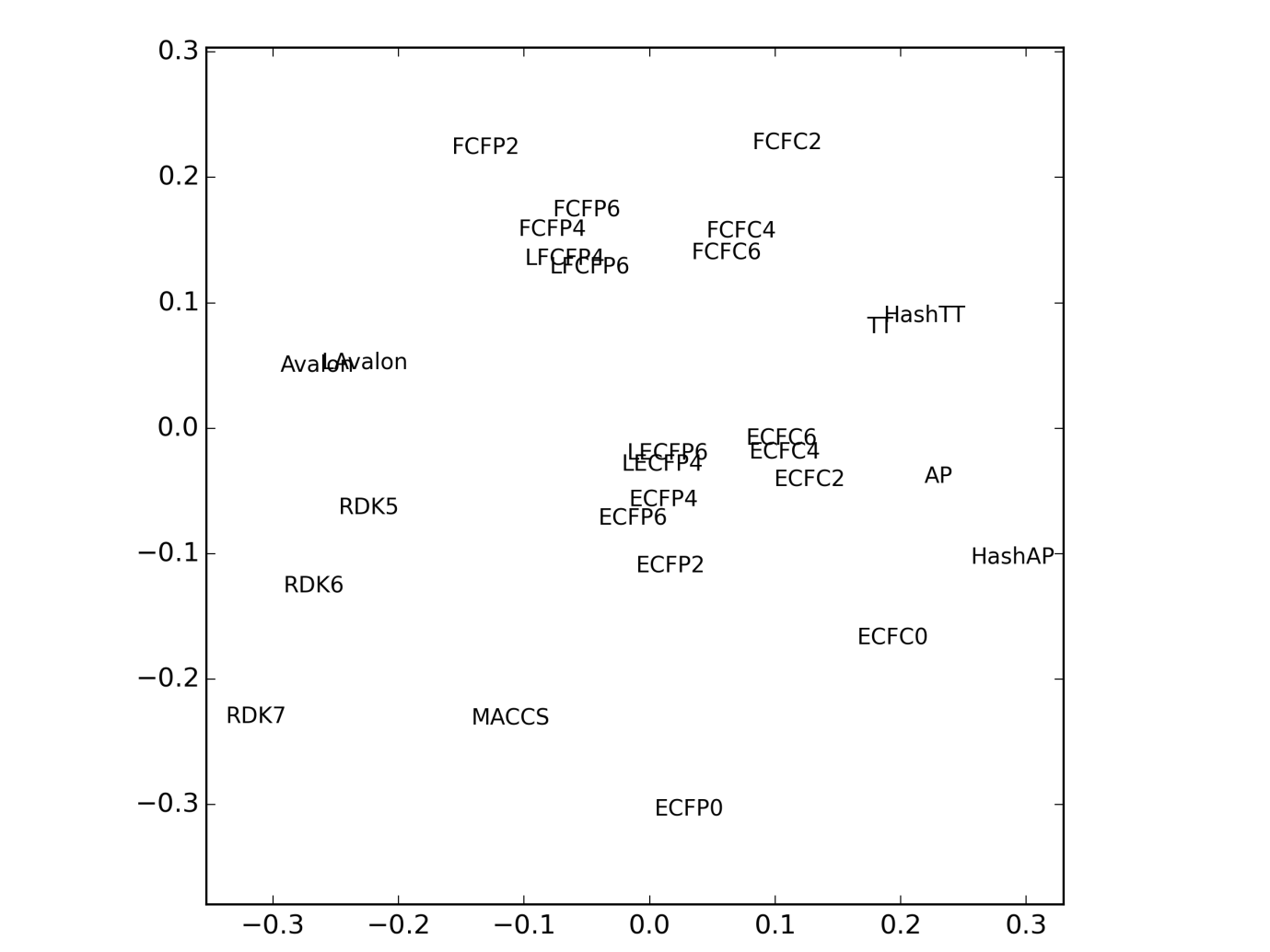


**Figure S4 – 2D visualisation of the similarity of different fingerprints based on how they order the series in the multi-assay benchmark according to different fingerprints.** A distance matrix was created from the rank correlations between the orderings by subtracting them from 1.0. Multidimensional scaling was used to visualise this distance matrix in 2D.
